# Efficacy and safety of abdominal acupuncture for knee osteoarthritis: A protocol for systematic review and meta-analysis

**DOI:** 10.1097/MD.0000000000023628

**Published:** 2021-04-16

**Authors:** Min Liu, Meinian Liu, Haitao Zhang, Guanrong Peng, Xiaobo Sun, Xingyang Zhu, Yirong Zeng

**Affiliations:** a The First Clinical Medical School, Guangzhou University of Chinese Medicine, Guangdong; b The Affiliated Hospital of Jiangxi University of Traditional Chinese Medicine, Nanchang; c Department of Orthopaedics, The First Affiliated Hospital of Guangzhou, University of Chinese Medicine, Guangdong, China.

**Keywords:** abdominal acupuncture, knee osteoarthritis, protocol, randomized controlled trial

## Abstract

**Background:**

Knee osteoarthritis (KOA) is a disease based on degenerative pathological changes. Most commonly seen in the elderly and is one of Kenn's leading causes, its symptoms include swollen knees, pain in walking up and downstairs. If left untreated, it can lead to joint deformity and disability. Many clinical studies have reported that abdominal acupuncture has a good effect on KOA treatment, but there is no relevant systematic review. So the purpose of this study is to evaluate the effectiveness and safety of abdominal acupuncture in treating KOA.

**Methods:**

The following 8 electronic databases will be searched, including PubMed, Embase, the Cochrane Library, China National Knowledge Infrastructure (CNKI), Web of Science, Chinese Scientific Journal Database (VIP), Wanfang Database, and Chinese Biomedical Literatures Database (CBM) from their inception to November 1, 2020 without any restrictions. Researchers retrieve the literature and extracted the data, evaluation of research methods, quality of literature. The outcomes will include a Visual Analogue Scale. The Western Ontario and McMaster Universities Osteoarthritis Index, total effective rate, incidence of any adverse events. We use the Cochrane Risk of a bias assessment tool to evaluate methodological qualities. Data synthesis will be completed by RevMan 5.3.0.

**Results:**

We will show the results of this study in a peer-reviewed journal.

**Conclusions:**

This meta-analysis will provide reliable evidence for abdominal acupuncture treatment of KOA.

**INPLASY registration number:**

INPLASY2020110020.

## 1 Introduction

Knee osteoarthritis (KOA) is a local inflammation and chronic strain of the knee joint that causes degeneration of the articular surface cartilage and reactive bone loss of the subchondral bone plate, a range of signs and symptoms in the knee.^[1]^ The disease is more prevalent in people older than 65 years of age, and there appears to be a trend toward increased incidence in younger age groups.^[2,3]^ The onset of KOA is related to many factors, including gender,^[4,5]^ age,^[6]^ genetic factors,^[7,8]^ physical factors.^[9,10]^ Global age-standardized prevalence of knee osteoarthritis is reported to be 3.8%, with women predominating.^[11]^ The Global Burden of Disease Study, conducted in 2010, ranked hip and knee arthritis (OA) as the 11th most disabling of 129 musculoskeletal disorders.^[12]^ Clinical features of KOA include joint pain, stiffness, claudication, and limited range of motion, resulting in functional limitations in walking, squatting, and sitting and standing activities.^[13]^ KOA is associated with pain and disability, high treatment costs, decreased productivity, and an increasing burden on society.^[12]^

The current main treatments include intra-articular glucocorticoid injections,^[14]^ NSAIDs, exercise, and (if appropriate) weight loss,^[15]^ a high success rate has been shown in the treatment of KOA with intra-articular glucocorticoid injections, However, benefits may be short-lived, and adverse effects on the joint have been reported, including a small increase in loss of cartilage volume of uncertain clinical relevance.^[16]^ Abdominal acupuncture, as a complementary and alternative therapy, has been developed in China for thousands of years. Abdominal acupuncture has a relatively good effect in the treatment of KOA.^[17]^ A large number of clinical studies on the treatment of KOA with abdominal acupuncture have been reported, but there is no relevant systematic review. So this research aims to systematically and comprehensively evaluate the safety and efficacy of abdominal acupuncture in the treatment of KOA.

## 2 Methods

### 2.1 Study registration

This protocol was registered with the International Platform of Registered Systematic Review and Meta-Analysis Protocols (INPLASY) on November 5, 2020 (registration number INPLASY2020110020). It could be obtained from https://inplasy.com/inplasy-2020-11-0020. This report will be conducted based on the preferred reporting items for systematic reviews and meta-analyses protocols (PRISMA) statement guidelines.^[18]^

### 2.2 Inclusion criteria

#### 2.2.1 Study type

All randomized controlled trials (RCT) and quasi-RCTs study on abdominal acupuncture in the treatment of KOA will be included.

#### 2.2.2 Participants

All patients included in the study were diagnosed with KOA, regardless of age, gender, race.

#### 2.2.3 Type of intervention

The trail group uses abdominal acupuncture or combination therapy with other treatments. The acupuncture method, acupoint selection, and needles are not limited; the control group uses the intra-articular glucocorticoid injections, and other adjuvant treatments can be appropriately added. The trail group and the control group are not limited in terms of medication, dosage, and treatment course.

#### 2.2.4 Type of outcome measures

##### 2.2.4.1 Primary outcomes

Primary outcome measures will include:

1. The effective rate.
2. Visual Analogue Scale.

##### 2.2.4.2 Secondary outcomes

Secondary outcome measures will include:

1. Western Ontario and McMaster Universities Osteoarthritis Index.
2. Incidence of any adverse events.

### 2.3 Exclusion criteria

The following exclusion criteria are presented:

1. The same study or duplicated publications.
2. Case report.
3. Theoretical or basic research.
4. Unable to get available data through various means.

### 2.4 Search strategy

The following 8 electronic databases will be searched, including PubMed, Embase, the Cochrane Library, China National Knowledge Infrastructure (CNKI), Web of Science, Chinese Scientific Journal Database (VIP), Wanfang Database, and Chinese Biomedical Literatures Database (CBM) from their inception to November 2020 without any restrictions. We strictly follow the PRISMA^[19]^ statement, The research only includes human subjects, The main search terms: “abdominal acupuncture,” “Osteoarthritis, Knee,” and “randomized controlled trial.” will be included. We use similar search strategies for all electronic databases. We will also search for eligible trial, which is unpublished or ongoing. The search strategy for PubMed is shown in Table 1.

**Table 1:** The search strategy used in the PubMed database.

Search	Query
#1	“osteoarthritis, knee” [MeSH Terms]
#2	(((Knee Osteoarthritides [Title/Abstract]) OR (Knee Osteoarthritis [Title/Abstract])) OR (Osteoarthritis of Knee [Title/Abstract])) OR (Osteoarthritis of the Knee [Title/Abstract])
#3	#1 or #2
#4	“Acupuncture” [MeSH Terms]
#5	“needling” [Title/Abstract]
#6	#4 or #5
#7	“abdominal” [Title/Abstract]
#8	randomized controlled trial [Publication Type] OR randomized [Title/Abstract] OR placebo [Title/Abstract]
#9	#3 and #6 and #7 and #8

### 2.5 Study selection

**Figure 1:**
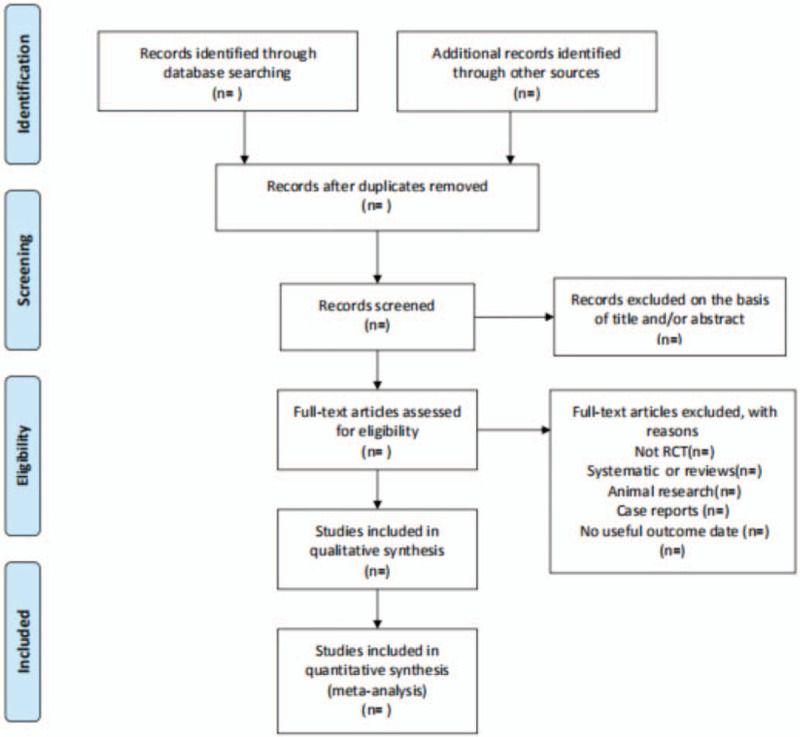
Flowchart of literature selection.

All literature retrieved from electronic databases will be imported into NoteExpress 3.2.0 software for category management, excluding double-checked and published literature. All researchers will discuss and define the selection criteria before selecting the literature. Two reviewers will independently assess the retrieved studies against the inclusion criteria. In the initial selection of studies, only titles and abstracts will be reviewed to exclude inappropriate publications. Studies that do not match will be removed to the trash folder in the software. Reasons for exclusion will be recorded as an Excel dataset. The next step will be to further evaluate the included studies by reading the full text. Two reviewers will check the reference list to identify trials that may have been missed. Two reviewers will cross-check the screening results. The reviewers, if they disagree, will resolve their disagreement by consensus. Any disagreement will be resolved by discussion between the 2 authors and the third author, arbitration, if necessary. The above process will be presented in the form of a PRISMA flowchart. (Fig. 1)

### 2.6 Data extraction and management

Data will be extracted independently from a pre-established data extraction table. The following basic information will be extracted: author, year, country, sample size, mean age, randomized method, intervention, control measures, follow-up time, outcome measures, and adverse effects. Three reviewers will cross-check the results. They are (Min Liu, Meinian Liu, and Haitao Zhang). If there is any disagreement, it will be negotiated or arbitrated by the fourth investigator (Yirong Zeng). Besides, the intention-to-treat analysis of this study will be used to address missing data. Data. NoteExpress 3.2.0 and Excel 2007 software will be applied. To extract the eligible data. When vital data in the study are incomplete or missing, we will have the option to contact the first author or the corresponding author to obtain the data by phone or email.

### 2.7 Assessment of risk of bias in included studies

The risk of bias in the included studies will be assessed by 2 independent reviewers (Min Liu and Meinian Liu) using the assessment tool of Cochrane Reviewer's Handbook 5.0.24.^[20]^ We will assess the risk of bias in the following areas: allocation sequence generation, allocation sequence concealment, blinding of personnel and outcome assessors, incomplete information. Outcome data, selective reporting of outcomes, and other sources of data bias. The assessment will be divided into 3 levels: low risk, high risk, and unclear risk. Ambiguous items in the study will be queried by Contact the appropriate author for details. Any disagreement will be resolved through discussion with the third reviewer (Yirong Zeng).

### 2.8 Data synthesis

#### 2.8.1 Measures of treatment effect

We will use RevManV.5.3.0 software for data analysis and quantitative data synthesis. Dichotomous data will be analyzed using risk ratios with 95% confidence intervals (95% CI). For continuous outcomes, we will analyze using the mean difference or standardized mean difference with 95% CI,^[21]^ when fewer than 2 studies are included for each outcome measure, only descriptive analyses will be performed to summarize the results.

#### 2.8.2 Heterogeneity analysis

Statistical heterogeneity between included studies will be assessed strictly according to the criteria (*P* > .1 and *I*^2^ < 50%) and displayed as a forest plot. When *P* > .1 and *I*^2^ < 50%, lower heterogeneity was analyzed using a fixed-effects model; when *P* < .1 and *I*^2^ > 50%, higher heterogeneity was analyzed using a random-effects model. When the included studies’ heterogeneity is significant, we will choose subgroup analysis or sensitivity analysis to search for possible sources clinically and methodologically.

#### 2.8.3 Subgroup analysis

If sufficient data are available, we will conduct subgroup analyses based on the following themes: age, duration of treatment, study quality, type of intervention in the control or study group, and so on.

#### 2.8.4 Sensitivity analysis

Sensitivity analysis will be used to validate the robustness of the review findings. We will consider several decision points in the systematic review process to implement the sensitivity review, such as sample size, missing data results, and methodological quality. Besides, the analysis will be repeated after excluding studies with low methodological quality.

#### 2.8.5 Publication bias

When the number of eligible RCTs is γ 10,^[22]^ publication bias will be detected using a funnel plot developed by Egger test.

#### 2.8.6 Grading the quality of evidence

A summary table of findings will be generated and included in the final report. Three investigators will assess the quality of each selected study through a tiered proposal assessment, development, and evaluation methodology. The following areas will be assessed: risk of bias, consistency, directness, precision, publication bias, and additional scores. There will be 4 levels of assessment: high, medium, low, and very low quality.^[23]^

#### 2.8.7 Ethics and dissemination

Ethical approval is not required as data from individual patients are not included, and there are no privacy implications. We will disseminate the results of this systematic review by publishing the manuscript in a peer-reviewed journal or presenting the relevant conference findings.

## 3 Discussion

KOA is one of the widespread clinical diseases. With the increase of age and incorrect exercise mode, more and more people are suffering from it. The main symptom is pain in the knee, which affects people's learning and life. Currently, the primary conservative treatment including intra-articular glucocorticoid injections, tui na, massage, and so on, When conventional therapy does not work, surgery can also be used, but people are often reluctant to accept surgical treatment because the surgery has certain risks and the possibility of failure. Abdominal acupuncture has been developed in China for thousands of years as a traditional healing method. The main effect is to invigorate blood circulation and eliminate stasis, warming the meridians. There are many clinical reports of using abdominal acupuncture to treat KOA, and it has the characteristics of sound effect, low cost, short course, low recurrence rate, and few side effects. However, there is a lack of systematic reviews and meta-analyses of abdominal acupuncture treatment for KOA. Therefore, we intend to demonstrate the efficacy and safety of abdominal acupuncture for KOA through this systematic review and meta-analysis. Finally, we hope that the results of this review will provide clinicians with more reliable, evidence-based evidence for the treatment of KOA.

## Author contributions

**Conceptualization:** Min Liu, Yirong Zeng.

**Data curation:** Min Liu, Meinian Liu, and Haitao Zhang.

**Formal analysis:** Min Liu, Meinian Liu, and Haitao Zhang.

**Investigation:** Yirong Zeng, Min Liu.

**Methodology:** Guanrong Peng, Xiaobo Sun.

**Software:** Meinian Liu, Xingyang Zhu.

**Supervision:** Xingyang Zhu, Yirong Zeng.

**Writing – original draft:** Min Liu, Meinian Liu, and Haitao Zhang.

**Writing – review & editing:** Yirong Zeng, Guanrong Peng, Xiaobo Sun, Xingyang Zhu.
